# Effects of S100B on Serotonergic Plasticity and Neuroinflammation in the Hippocampus in Down Syndrome and Alzheimer's Disease: Studies in an S100B Overexpressing Mouse Model

**DOI:** 10.1155/2010/153657

**Published:** 2010-08-22

**Authors:** Lee A. Shapiro, Lynn A. Bialowas-McGoey, Patricia M. Whitaker-Azmitia

**Affiliations:** ^1^Departments of Surgery, Neurosurgery, and Neuroscience and Experimental Therapeutics, Texas A&M Health Science Center, College of Medicine, Scott & White Hospital, Central Texas Veterans Health System, Temple, TX 76504, USA; ^2^Program in Biopsychology, Department of Psychology, State University of New York, Stony Brook, NY 11794-2500, USA; ^3^Department of Psychology, Dowling College, Oakdale, NY 11769, USA

## Abstract

S100B promotes development and maturation in the mammalian brain. However, prolonged or extensive exposure can lead to neurodegeneration. Two important functions of S100B in this regard, are its role in the development and plasticity of the serotonergic neurotransmitter system, and its role in the cascade of glial changes associated with neuroinflammation. Both of these processes are therefore accelerated towards degeneration in disease processes wherein S100B is increased, notably, Alzheimer's disease (AD) and Down syndrome (DS). In order to study the role of S100B in this context, we have examined S100B overexpressing transgenic mice. Similar to AD and DS, the transgenic animals show a profound change in serotonin innervation. By 28 weeks of age, there is a significant loss of terminals in the hippocampus. Similarly, the transgenic animals show neuroinflammatory changes analogous with AD and DS. These include decreased numbers of mature, stable astroglial cells, increased numbers of activated microglial cells and increased microglial expression of the cell surface receptor RAGE. Eventually, the S100B transgenic animals show neurodegeneration and the appearance of hyperphosphorylated tau structures, as seen in late stage DS and AD. The role of S100B in these conditions is discussed.

## 1. Introduction

S100B is a member of the EF-hand type of calcium binding S100 protein family which consists of approximately 20 different proteins. S100B is the only member found on chromosome 21, the remaining largely being found in a cluster on chromosome 1. S100B is the principle S100 found in brain, and makes up approximately 0.5% of all brain proteins. Under normal physiological states, S100B is expressed predominantly in astroglial cells of the central nervous system (CNS), and also to a lesser extent, in neurons, microglia, and oligodendrocytes [1–7]. However, in neuropathological conditions, including those induced by environmental stressors, infection, ischemia, trauma, psychiatric conditions such as depression [8–10], and schizophrenia [11, 12], the cellular and tissue distribution of S100B within the brain may change. As a brain-derived protein measurable in peripheral samples, S100B is often used as a biochemical marker for brain injury. In the periphery, S100B is expressed by Schwann cells, ependymocytes, adipocytes, chondrocytes, melanocytes, dendritic cells, skeletal muscles, and myocardium [13]. The fact that S100B is increased in a wide variety of pathological conditions is indicative of the diverse functions that this protein plays throughout the body and brain [14, 15]. 

Donato has suggested that S100B is important for the progression of cells through the cell cycle [16, 17]. We have used the term, “accelerated aging,” to describe cell cycle changes in the CNS. Regardless of the term employed, the message is the same: S100B acts in the brain to promote development and aid in recovery, but also as an inflammatory protein with a role in aging and neuropathology. 

Serum levels of S100B in humans are age-dependent [18] being highest in newborn, stable throughout adulthood and increasing again in aging. A similar profile is seen in rodents [19]. It has been suggested that the increased S100B in aging is related to a lifetime of proinflammatory events, including ischemia, trauma, and infections. The effects of S100B in brain are not only age-dependent, they are also concentration-dependent, such that it is protective and trophic at low concentrations [20], but toxic and proapoptotic at high concentrations [21, 22]. 

The neurobiological effects of S100B are known to occur intracellularly, in the cells which express the proteins, as well as extracellularly, as the protein can be released, notably in response to serotonin (5-HT) binding to the 5-HT1A receptor [16, 17, 23]. In addition to 5-HT, other factors known to stimulate S100B release include IL-6, adenosine, glutamate and cannabinoid receptors [24–26]. There is also a substantial amount of passive release into the neuropil. 

Some of the detrimental extracellular effects of S100B may be mediated via the cell-surface receptor for advanced glycation end-products (RAGE). In the central nervous system, RAGE can be localized to neurons, microglial cells, and astrocytes. The RAGE receptor is a member of the immunoglobulin superfamily and leads to cellular dysfunction in a number of disorders. This receptor was originally identified and characterized for its binding of advanced glycation end-products (AGEs) which accumulate in diseases such as diabetes and renal failure [27]. More recently, RAGE was shown to be a multiligand, cell-surface receptor, responding to a number of ligands, including S100B. It is interesting to note that increased S100B results in increased RAGE [28]. Consistent with this notion is the fact that during development, or following an insult, both S100B and RAGE increase whereas in normal adult tissue, relatively low RAGE expression is found. 

Elevated brain S100B expression occurs in various disease states, including Alzheimer's disease (AD) and Down syndrome (DS). The gene for S100B is found on chromosome 21 and is often part of the triplicated chromosome in DS. Interestingly, Down Syndrome almost inevitably leads to an extremely early development of AD and the increase in S100B is thought to contribute to the pathology of both. Although S100B has a variety of cellular effects throughout the body and brain, its role in neuroinflammation, and in the regulation and maintenance of the serotonergic nervous system is highlighted, with a particular focus on the hippocampus. Understanding neuroinflammation and serotonin neuronal plasticity in this brain region may help to explain many findings of changes in learning and memory, as well as the occurrence of depression symptoms. This is especially pertinent when considering co-morbidity in conditions wherein the levels of S100B are altered, such as AD and DS.

### 1.1. Alzheimer's Disease

Numerous human postmortem studies have shown increased S100B in the brain of subjects with AD [29–31]. This elevated S100B correlates with many of the neuropathological changes, including the presence of beta-amyloid plaques and neurofibrillary tangles. Elevated S100B has also been found in the cerebrospinal fluid of AD patients [32] and correlates with the degree of brain atrophy [33]. In a transgenic mouse model of amyloid pathology, increasing S100B has been shown to increase plaque formation [34]. Little work has been done on the role which S100B overexpression might directly play in the formation of neurofibrillary tangles. However, one recent study showed that S100B acts through the RAGE receptor to result in hyperphosphorylated tau, a contributor to neurofibrillary tangles [35]. In the current review, data have been included showing hyperphosphorylated tau in S100B overexpressing mice. Thus, chronic overexpression of S100B may lead to both plaque and tangle formation.

### 1.2. Down Syndrome

Serum levels of S100B are greatly increased in Down syndrome [36] and postmortem brain studies show lifelong overexpression of the protein [37, 38]. As in AD, many of the neuropathological changes in DS are thought to be related to the overexpression of S100B [39, 40]. Previous studies have shown behavioral, neuropathological and cardiovascular alterations in a transgenic mouse overexpressing human S100B protein which we use in our studies [40–46]. These mice were designed to model the elevated S100B that is often a part of the Down syndrome genotype [47]. Similar to DS patients who almost inevitably show premature signs of aging and AD, the S100B mice show signs of accelerated aging [48], neuropathology [44, 46, 49], and behavioral deficits [42, 43]. Thus, the S100B transgenic mouse is well-suited to study the influence of chronic S100B over-expression on brain and behavior in the context of DS, including the accelerated development of AD neuropathology.

## 2. S100B and Serotonin Neuroplasticity

The neurons which produce serotonin are amongst the earliest developing neurons in the mammalian brain and serotonin plays a role in the development and maturation of many brain regions [50]. Serotonin also becomes the most widely-distributed system throughout the cortex, touching on virtually every neuron in cortex [51]. This widespread distribution and early development allows for a role for serotonin in maintaining and promoting synaptic plasticity. Much of this effect of serotonin is mediated through the release of S100B. During development, S100B promotes process outgrowth from cortical cells [52, 53] as well as promoting dendritic development in the hippocampus [54]. A reciprocal relationship occurs, whereby serotonin (through 5-HT1A receptors) not only releases S100B [55, 56], but S100B also promotes development of serotonin terminals. Treatment with drugs and agents which increase serotonin, such as MDMA [57], or selective serotonin reuptake inhibitors [19, 58], increase the expression of S100B. Thus, S100B levels are important in regulating the terminal outgrowth and maintenance of serotonin terminals. 

Interestingly, by twenty years of age, PET studies in normal populations have shown that the serotonin transporter begins to decrease and continues to do so at the approximate rate of 10% per decade up until the 8th decade [59]. A further loss with aging may increase if S100B increases. For example, in neurodegenerative disease, such as Parkinson's disease and frontal lobe dementia, the forebrain serotonin fibers are decreased and become dystrophic, with enlarged and bulbous endings [60].

In midgestation DS fetuses, there is a significant (40%) loss of serotonin content in frontal cortex [61], although there is no loss of terminal development [62]. As subjects age, pronounced region-specific changes in serotonin terminal areas are seen. In the adult, loss of serotonin content is seen in caudate and temporal cortex, but increases are found in occipital cortex [63]. Studies of the serotonin terminal density, show that adult DS have increased terminals in frontal cortex [64]. Unfortunately, there are no reported studies on serotonin content or terminal density in hippocampus of DS, of which we are aware. 

The increased levels of S100B in Alzheimer's disease may also be associated with a loss of serotonin. Neurofibrillary tangles have been shown to occur in the raphe nuclei, the site of serotonergic cell bodies [65, 66], and there is a loss of serotonin terminals in hippocampus and several other subcortical and cortical structures [67–71].

**Figure 1:**
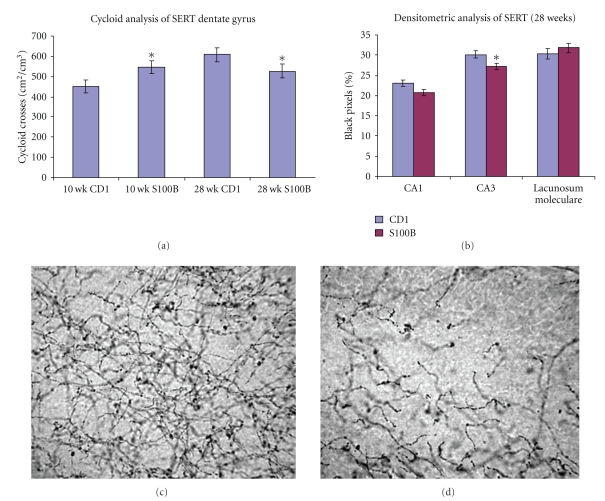
Serotonergic fiber analysis in the hippocampus of normal and S100B transgenic mice. In (a), a graph of the mean cycloid crosses (cm^2^/cm^3^) is shown to indicate the results from the stereological analysis showing a significant increase (*P* < .05) of SERT fibers in the dentate gyrus of 10-week-old S100B transgenic mice. Alternatively, at 28 weeks, significantly less (*P* < .05) SERT fibers were observed in the dentate gyrus of S100B transgenic mice. In (b), the results from the densitometric analysis are shown in areas CA1, CA3, and Lacunosum Moleculare of the hippocampus from 28-week-old mice. Note that there is a significant decrease (*P = .027) in the density of serotonergic fibers in the S100B transgenic mice. It is pertinent to note that this densitometric method was verified in the infrapyramidal blade, where the results were consistent with the stereological analysis. There were no significant differences at the 10 week timepoint (data not shown) in CA1, CA3, or Lacunosum Moleculare. In (c) and (d), representative photomicrographs from the infrapyramidal blade of 28-week-old control (c) and S100B transgenic (d) mice. Note the apparent decreased density of serotonergic fibers in the S100B transgenic mice (d).

We have examined the development and maintenance of serotonin terminals in the S100B overexpressing transgenic mouse using an antibody raised against the serotonin transporter (SERT) which stains serotonergic fibers. Our results show increased serotonergic fibers in the dentate gyrus of the hippocampus of 10 week old S100B transgenic mice. Conversely, at 28 weeks, there is an accelerated loss of SERT-stained serotonin fibers as these animals age (Figure 1).

**Figure 2:**
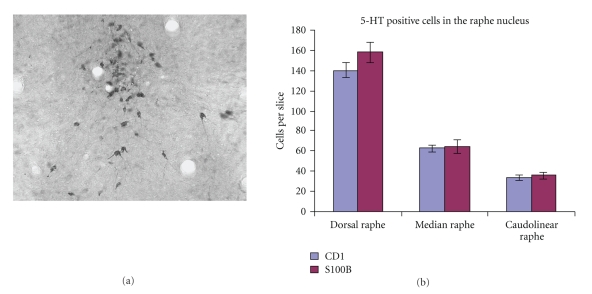
Serotonin cells in the raphe nucleus of 7-month-old control and S100B transgenic mice. In (a), a representative photomicrograph is shown to illustrate the 5-HT immunohistochemical labeling of serotonin cells in the raphe nuclei. In (b), a graph of the means showing no significant differences in the number of serotonin neurons between CD-1 control and S100B transgenic mice in the dorsal, median, and caudolinear segments of the raphe nucleus. It is pertinent to note that when all three regions were combined, the data were still not significantly different (data not shown).

Despite the decrease in serotonergic fibers, stereological analysis found no evidence for a decreased number of serotonin neurons in the raphe nucleus, as in the human post-mortem studies of AD (Figure 2). A recent autoradiographic study found increased serotonin terminal density in the substantia nigra, but not the caudate of S100B overexpressing animals [72]. Therefore, changes to serotonin terminals in S100B overexpressing mice appear to depend on the age of the animals and on the structures examined, very much as has been observed in human DS tissue. In the two regions studied here, the findings suggest a loss of serotonin neuroplasticity in the memory center, the hippocampus, but no changes in the motor region of the caudate, which would correlate with the human cases. 

These findings show that changes in S100B could lead to the changes in serotonin observed in DS and AD. Moreover, since serotonin has long been known to be related to depression, our findings may imply a role for S100B in depression. S100B levels are increased in CSF and serum of patients with depression [73, 74], and the best response to therapy is predicted by the highest levels of S100B [75]. Selective serotonin re-uptake inhibitors are a mainstay of treatment for depression and infants exposed prenatally to SSRI's have lower levels of S100B [76]. This may prove to be a serious teratogenic effect, given the role of S100B and serotonin in brain development.

## 3. Neuroinflammation

A combination of natural aging and numerous intermittent peaks in inflammatory processes, caused by environmental stressors such as infection, ischemia, or toxins, may ultimately lead to the pathological changes associated with aging. In the brain, these processes are termed neuroinflammation and generally refer to those processes known as “reactive gliosis.” That is, the accumulation of enlarged or dystrophic microglial and astroglial cells [77]. Neuroinflammation can then lead to loss of neurons and loss of brain functions in a variety of neurodegenerative states [78, 79]. 

The role of S100B in neuroinflammation is becoming increasingly evident [19, 80]. S100B is predominantly expressed in astroglial cells of the mammalian nervous system, but during neuroinflammatory states, the protein can also be found in microglia [6], oligodendrocytes [1, 2], radial glia [7], and different classes of neurons [3, 5, 81]. In DS, activated astrocytes are already observed in the prenatal brain and are increasingly found with age. These increases correlate with the number of beta-amyloid plaques [37, 38]. Activated astrocytes are also found in AD [79] and S100B increases may play a role in their appearance. Activated microglial cells and elevated IL-6 are observed to correlate with elevated S100B in both disorders [82, 83]. In DS fetuses, microglial outnumber astroglia, which is not usually the case in normal fetuses [84]. Thus, chronic inflammation occurs in DS and may be involved in the inevitable, early development of AD. 

In the S100B overexpressing mice, we have looked for effects of S100B on astroglial and microglial cells, in order to confirm the role of S100B in the neuroinflammatory changes seen in DS and AD. Finally, we have looked at markers of degeneration. The results of these studies are described in the following pages.

### 3.1. Astroglia

Astroglial appearance can generally be characterized by number, cell body size, and type of processes. Chronic overexpression of S100B results in changes to the number and morphology of S100B-labeled astrocytes at early (12 weeks) and later (28 weeks) timepoints. In the S100B overexpressing mice, astroglial cells are rarely observed to have the complex morphology with numerous processes, which are seen in control mice. Within astroglial cells, S100B regulates Ca^2+^ levels and once activated by Ca^2+^, S100B interacts with intermediate filaments including GFAP and vimentin [85, 86]. This interaction leads to inhibition of filament polymerization, resulting in changes to the cytoskeleton and altered astroglial morphology [87–89]. Thus, in the S100B overexpressing animals, the excess S100B could lead to pronounced inhibition of intermediate filament polymerization and thus an instable cytoskeleton that lacks multiple processes. As the S100B transgenic mice age, the number of mature, multiprocessed S100B-labeled astrocytes is noticeably decreased (Figure 4). Moreover, as the transgenic animals mature, the atypical astrocytic morphology is more pronounced, with relatively larger cell bodies. This morphology is not observed in the control animals at either age (see Figure 4).

**Figure 3:**
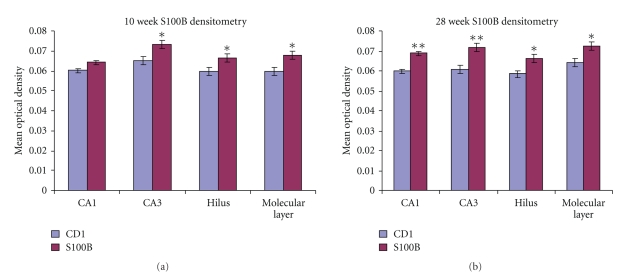
Immunodensitometry of S100B at 10-and 28-weeks- in CD1 control and S100B transgenic mice. In (a), the S100B mice shown significantly (*P < .05) elevated S100B in CA3 stratum radiatum, the hilus, and molecular layers of the dentate gyrus. There is also a trend towards significance in CA1 stratum radiatum. In (b), all of these regions are significantly elevated (*P < .05; **P < .01) at the 28 week time point.

**Figure 4:**
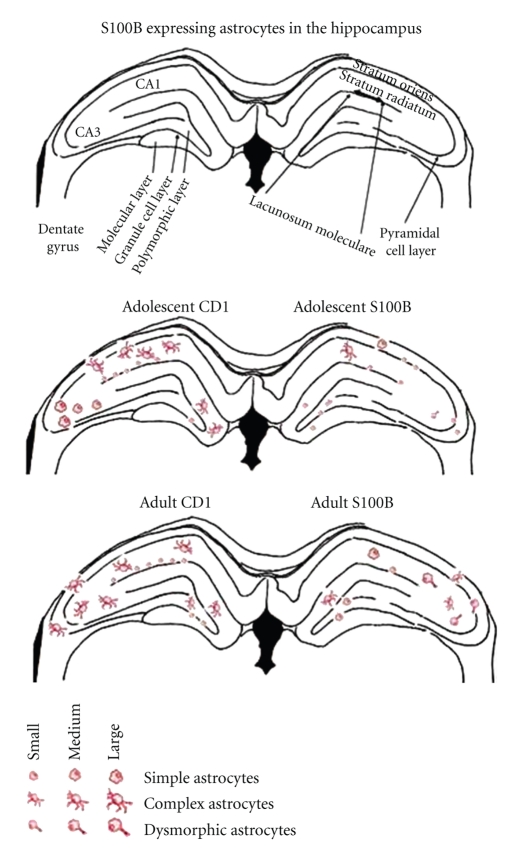
Schematic diagram summarizing the distribution analysis of S100B-labeled astrocytes in the hippocampus of 10 and 28-week-old CD-1 control and S100B transgenic mice. *10 weeks*: CD 1 animals showed a high density of large complex cells in CA1 SR, and a large number of small, simple cells in CA1 LM. Although similar cell types occurred in these regions of the S100B mice, their numbers were markedly reduced. In CA3, CD1 mice had a large number of medium to large simple cells, while the S100B mice again showed far fewer cells, which were smaller simple cells. In addition, there were a number of small atypical cells. Again, in the dentate gyrus, the adolescent CD-1 animals showed predominantly medium-sized complex cells, while the S100B animals showed small, simple cells. Occasionally, the S100 animals had small atypical cells within the polymorphic region of DG, which were virtually absent from the CD-1 mice. The cells of the S100B animals showed pronounced “haloes” of tissue S100B-IR around all cell types of all regions, suggesting large amounts of S-100B release. A representative photomicrograph of these findings is given in Figure 5. *28 weeks*: As the CD-1 animals matured, the number of complex cells in CA1 SR decline, but there continue to be a large number of small simple cells in LM. In CA3, the cells have become more complex. The dentate gyrus shows fewer complex cells, more simple cells, and now the occasional complex cell is seen within the polymorphic region. In the S100B animals, there continues to be an overall decrease in S100B-IR cells throughout the hippocampus, compared to adult CD 1 mice. The atypical cells first seen in adolescence are now larger and more numerous, particularly in CA3. These cells are virtually absent from the CD-1 mice at any age. CA1 cells are simple of mixed size, with an absence of complex cells. There are a small number of complex cells in CA3, more than in adolescent S100B animals, but smaller and fewer than in CD1 adults. Cells of the DG are predominantly medium sized simple and occasionally medium-sized complex. The atypical cells are not seen here. Again, large haloes (Figure 5) of S100B-IR are seen around the cells.

**Figure 5:**
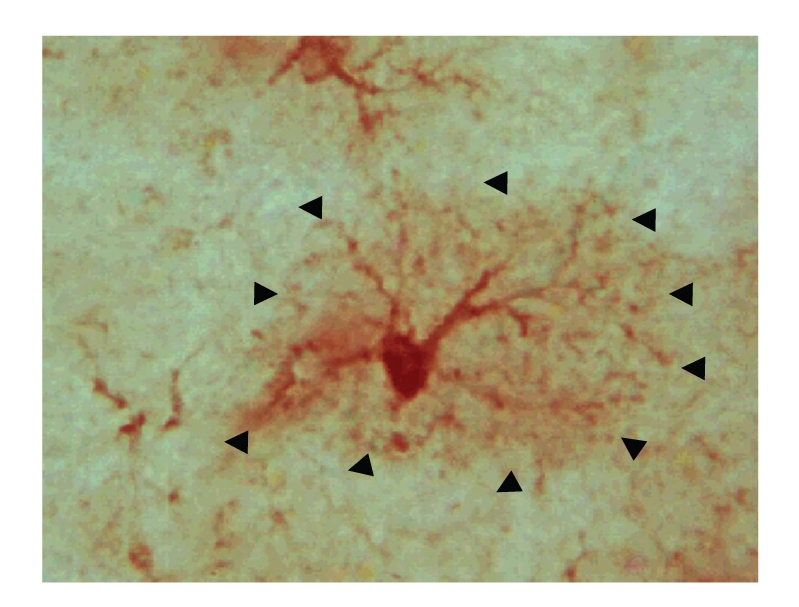
Photomicrograph of an S100B-positive astrocyte depicting a halo (outlined by arrowheads) of S100B-positive immunostaining around the astrocyte. Note that the majority of adult S100B+ astrocytes in the hippocampus of S100B transgenic mice display this type of appearance.

It is important to note previous studies showing that at low concentrations, S100B stimulates astroglial proliferation [90], and at high concentrations, the protein is toxic to astrocytes [21, 91]. Considering our findings showing that tissue levels of S100B are elevated throughout life in the transgenic animals (Figure 3), it is not surprising that the astroglial cells themselves are fewer in number and have an atypical morphology (Figure 4), changing with age. In addition to the changes in cell morphology, it is clear by the abundance of S100B in the parenchyma, that the astroglial cells are releasing relatively high amounts of S-100B into the surrounding neuropil. The released S100B is not evenly distributed throughout the neuropil, but rather is confined to characteristic “haloes” around the astroglial cell (Figure 5). Thus, chronic elevation of S100B in DS might be one mechanism whereby chronic astrocytic activation occurs. A second mechanism may be through the interaction between S100B and microglial cells.

### 3.2. Microglia

Microglial cells are the primary component of the brain's immune system and are a key part of neuroinflammatory processes [78]. As with astrocytes, microglial cells exhibit various morphologies that correlate with a continuum of functional states of resting, reactive, chronically active and phagocytotic [92]. When the brain is damaged, whether from seizures [93], trauma, or diseases such as multiple sclerosis, Creutzfeldt-Jakob, DS, and AD [94–97], microglial cells become activated [98]. Activated microglial cells proliferate [99], migrate to the site of injury [100], alter their morphology, and begin two important functions: initiation of inflammatory processes by release of inflammatory proteins [101–103] and phagocytosis [104]. 

**Figure 6:**
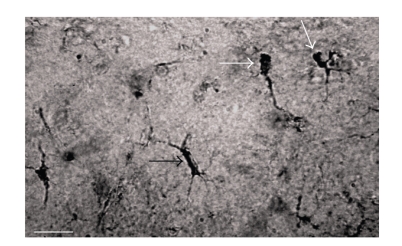
S100B-labeling of microglial cells in the hippocampus of 28-week-old S100B transgenic mice. In this micrograph, a rod-shaped cell (black arrow) is shown from an adult S-100B animal. This cell appears to be either an ependymal or microglial cell, based on descriptions by Hortega, (1920). Other cells (white arrows) in this photomicrograph appear to depict different stages of microglial differentiation. Scale  bar = 20 *μ*m.

Microglial activation is mediated by numerous substances released by the injured tissue [105] one of which is S100B [106]. Moreover, in pathological states, microglial cells express S100B [6] suggesting that they may be involved in a feedback loop. As can be seen in Figure 6, microglial cells labeled with an antibody to S100B exhibit various activated morphologies in adult S100B overexpressing mice. This is consistent with in vitro studies showing that S100B is expressed by a class of microglial cells [6]. Considering that microglial cells are also prominent features of DS fetuses [85], the data support the idea that S100B overexpression influences microglial cells during development and in adulthood.

S100B has been shown to have at least two effects on microglial cells, one of which (production of nitric oxide) is RAGE-independent whereas the other (increases in the transcription factor NFkappaB) is dependent on binding to microglial RAGE [107]. When S100B binds to RAGE, the microglial cells become activated. Thus, chronic S100B elevation may influence chronic microglial activation via this mechanism. Support for this idea is derived from the fact that binding of ligands to RAGE leads to activation of signaling pathways which in turn can modulate gene expression [108]. One such pathway activated is that of the proinflammatory transcription factor, NF*κ*B, which regulates cytokines, including interleukin IL-1, IL-6, and tumor necrosis factor [109, 110].

**Figure 7:**
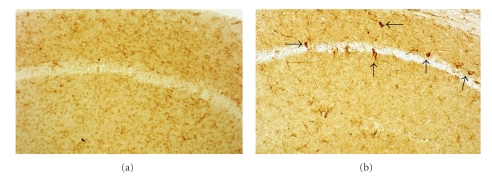
F4/80-labeling of macrophage/microglia in the CA1 pyramidal cell layer of 1-yr-old CD-1 and S100B transgenic mice. In CD-1 control animals (a), the labeling is found predominantly in resting microglial cells. Alternatively, in the S100B animals (b), several intensely labeled cells (arrows) are observed. These cells could be activated microglia, or another type of immune cell that may have infiltrated the hippocampus, such as macrophages or dendritic cells. 200X.

Both control and S100B-overexpressing animals show an increase in microglial cells and RAGE expression with age. However, this effect is more pronounced in the S100B animals, [46] (also see Figure 7). Thus, S100B binding to microglial RAGE may be a second mechanism whereby chronic S100B elevation in DS exacerbates chronic neuroinflammation.

### 3.3. Neuroinflammation and Neurodegeneration

**Figure 8:**
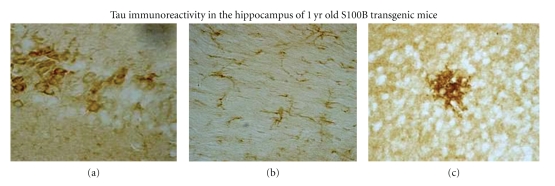
Tau-2-IR in the hippocampus of a one-year-old S100B transgenic animal. The antibody used in these studies recognizes a hyperphosphorylated form of tau usually associated with neurofibrillary tangles. In the developing brain, S100B promotes neurite outgrowth. However, in a brain chronically exposed to elevated levels of S100B, the neurites become dystrophic. (a) Cell body staining in pyramidal neurons of CA3, presumably to intracellular neurofibrillary tangles, 400X. (b) Neuropil threads in the lower blade of the dentate gyrus showing axonal accumulation of tau-2-IR, 600X. (c) Neuritic plaque in CA1 of stratum radiatum. This is a dense accumulation of neuritic fibers, similar to that described for this antibody in human AD subjects, associated with high levels of S100B (Sheng et al. [82]), 600X.

Although a role linking S100B directly to cell death in DS and AD has not been established, there is evidence from animal data that this may indeed be the case [111, 112]. S100B transgenic animals show neuronal loss and increased expression of the proapoptotic protein clusterin [48] which is also increased in hippocampus and frontal cortex of AD [113]. In addition, previous animal studies have shown that using arundic acid to negatively regulate S100B will ameliorate beta-amyloid deposits, plaques, and glial (astrocyte and microglial) hypertrophy [114]. Interestingly, recent findings in the S100B transgenic mice show that S100B overexpression can lead to increases in the hyperphosphorylated tau protein found in neurofibrillary tangles (Figure 8). In a mouse model of AD, chronic over-expression of S100B has been shown to intensify gliosis and amyloidosis [115]. Moreover, chronic over-expression of human S100B increases brain damage and peri-infarct gliosis after focal ischemia. Together, these data support the idea that high levels of S100B can be detrimental in neuropathological conditions. Considering these findings, the S100B transgenic mice could be used to provide insight into possible prevention and treatments, for plaques, tangles, or other neuropathologies associated with S-100B overexpression. 

For example, Vitamin E was examined in the S100B overexpressing mouse to determine if antioxidant treatment had the potential to attenuate neuroinflammatory damage associated with increased S100B. The results show that although Vitamin E decreased microglial activation in the control animals, it actually increased microglial activation and RAGE expression in S100B overexpressing mice [46]. We and others [53, 74] have hypothesized that in the state of chronic overexpression of S100B, oxidation of S100B serves to preferentially induce the neurotrophic properties over the neuroinflammatory processes. Thus, treatment with an antioxidant to an animal already over-expressing S100B may interrupt this feedback, leading to further increases in RAGE upregulation and glial activation. Vitamin E may appear to be beneficial in a control animal, but animals already overexpressing S100B may show an increase in neuroinflammatory processes as evidenced by increased microglial activation [46]. This study makes the important point that studying potential treatments to prevent neurodegeneration should be done in animals which resemble the human form of neuropathology.

## 4. Conclusion

In conclusion, the data demonstrate that chronic S-100B overexpression results in serotonergic fiber alterations in the hippocampus, but no alterations to the number of serotonin neurons in the raphe nuclei. The data also show that chronic S100B over-expression results in activated astrocytes and microglia beginning during neonatal development and persisting through adulthood. Such changes are associated with an increase in RAGE expression. Considering the appearance of TAU-immunolabeling as a result of chronic S100B overexpression, future studies are needed to better understand the role of S-100B in mediating serotonergic and glial alterations, and their role in neuropathologies, such as, DS, aging, and AD.

## Appendix

### A. Methods

#### A.1. Animals

Male S-100B transgenic mice, aged 10 weeks (*N* = 4–6) and 28 weeks (*n* = 4–6) were compared to age matched, 10-week-old congenic CD-1 (*N* = 4–6) and 28 week CD-1 (*N* = 4–6) control mice. The S-100B mice and their congenic CD1 controls used in this study, were raised in the animal care facilities at SUNY Stony Brook, under identical conditions, of 12/12 light/dark cycle with food and water provided ad libitum.

#### A.2. Immunocytochemistry

All animals were perfused with 50 mL of  .9% saline followed by 50 ml of 4% paraformaldehyde fixative. Brains were extracted and post fixed in 4% PLP for one hour. The brains were then cryoprotected successively in 10%, 20%, and 30% sucrose, each for twenty-four hours. Following cryoprotection, brains were sectioned on a freezing microtome at 40 um. Each group consisted of four to six male mice. For immunostaining, the tissue was first blocked in 1% peroxide for 1 hour and then blocked with 3% goat serum and  .03% triton-X, in PBS for 1 hour. The tissue is then incubated free floating, in primary antibody (S100B, Sigma; 5-HTT 1 : 1000, generous gift from Dr. Feng Zhou, University of Indiana; 5-HT, 1 : 200, sigma #S5545; F4/80, 1 : 1000, Serotec MCA497R; Tau2, 1 : 500, Sigma #T5530), with 3% normal goat serum and  .3% triton-X100, on a cold rotator (4C), for twenty four hours. Tissue was then rinsed in PBS and rotated for 1.5  hour, at room temperature, free floating in secondary IgG (Sigma, 1 : 200) raised against the appropriate species specific for each primary antibody, in PBS with 3% normal goat serum and  .3% triton-X100. Next, the tissue was rinsed in PBS and incubated in Vectastain elite avidin biotin complex (Vector, Burlingame, CA), for one hour, rotating at room temperature. Negative control sections were carried out simultaneously, following the same protocol, minus the primary antibodies. The antibody complex was visualized using  .05% diaminobenzadine (DAB), with 30% peroxidase added after the tissue soaked for 3 minutes. Tissue was rinsed and mounted onto gelatin coated slides, dehydrated in alcohol, cleared in xylene, and coverslips were applied with permount.

Slides were coded and all images were captured on an Olympus BH-2 microscope, with a Tripix digital camera. Images were saved on a windows-based computer. In all instances, data was analyzed by raters blind to the condition of the animals.

#### A.3. Serotonin Transporter Analysis

Estimates of the surface density (Sv) of SERTir fibers were obtained using the cycloid grid intercept method [116]. This method is based on Buffoun's needle principal [117], which states that the length of a object (a needle in Buffon's case, SERTir fibers in this experiment) is directly proportional to the number of random intersections it makes with lines on a grid and inversely proportional to the distance separating the lines on the grid. Mathematically this relationship is described as *P* = (2/pi)×(l/d), where *P* is the probability of an object-line intersection, 2/*p* *i* encompasses all possible angles of intersection between the object and the line, *l* is the length of the objectgh and *d* is the distance separating the lines on the grid. Cycloids are used in this experiment rather than a strait line grid, because in the case of the serotonin transporter fibers, the fibers are curved and transverse through multiple orientations of the three-dimensions of the tissue. Thus, the cycloid grid compensates for bias created by the cutting orientation [116]. For this experiment, a counting frame with 16 cycloid test lines, each with a length of 21 uM, was superimposed over photomicrographs of the infrapyramidal granule cell layer of the dentate gyrus, which was chosen as the horizontal orientation point. For each animal, the cycloid grid was superimposed over an area of molecular layer of the infrapyramidal blade of the dentate gyrus and intersections between the cycloid lines and SERTir fibers were counted. For each animal, 5 images separated by 240 uM were analyzed, corresponding to Paxino and Franklin plates 41–50 in the mouse brain atlas [118]. At the final magnification (600X), the total cycloid length for the grid was 336 *μ*M. Surface density (Sv) was calculated using the equation Sv = 2 × PL, where Sv is the surface area per unit volume of tissue, and PL is total number of intersections, divided by the total length of the probe. A fiber was considered to be in the plane of focus if it was not blurry. In order to insure the accuracy of scoring, three blind raters were given the same set of instructions for what constituted a cycloid intersection. The data were compared using regression analysis and once verified, the three scores for each image were averaged, to yield the average intersections per image. These averages are used to calculate Sv, which is expressed as CM^2^ of SERTir fibers, per CM^3^ of tissue (CM^2^/CM^3^). The average Sv for each animal was entered into SPSS (version 9.0) and analyzed using the univariate anova, with condition as fixed factor and surface density of SERT ir fibers, the dependant variable.

In addition to the stereological analysis of SERT fibers, we also performed a densitometric analysis of SERT containing processes using the University of Texas Image Tool program (V. 3.0). Using the threshold setting, the images were assigned a pixel value within which the SERT fibers would be transformed into black pixels and the surrounding neuropil would be transformed into white pixels. This method allowed us to identify only the fibers which stained intensely enough to fall within the threshold range, which were presumed to be those fibers within the range of the plane of focus of the objective lens (Olympus 60X, aperture =  .90). The number of black and white pixels was then quantified using the ImageTool program, which digitally automates the counting. For each animal, 5 images were examined from the infrapyramidal blade of the dentate gyrus and averaged, to yield a percentage of black pixels per animal. The averages for each animal were analyzed using the univariate Anova on SPSS (version 9.0).

This method proved to be reliable and consistent with the stereological probe technique in the dentate gyrus. Thus, additional photomicrographs were examined using this technique in the following hippocampal areas: CA1, CA3, stratum radiatum, and lacunosum molecular.

#### A.4. Analysis of 5-HT Cells in the Adult Raphe Nucleus

The staining protocol was as above, except, the primary antibody was incubated for forty-eight hours. Raphe nucleus sections, separated by 40 uM, containing 5-HTir neurons, were included for cell counts. Images were captured as stated above, and neurons were counted by a blind scorer. Neurons were counted in a 1.058 mm^2^ area, containing the dorsal, median, or caudolinear raphe nucleus. The sum of 5-HTir neurons counted for each region was divided by the sum of the number of images counted, to yield an average density of 5-HTir neurons in a 1.058 mm^2^ area. The data was analyzed using a multivariate analysis of variance, with condition as the fixed factor, and the dorsal, median, or caudolinear raphe nucleus as the dependant variables.

#### A.5. Detailed Morphological Analysis of S100B-Labeled Cells

Detailed morphological analysis of S-100B immunoreactive (IR) glial cells in the hippocampus was performed by raters blind to the age and genetic background of the animals. Within the hippocampus, the predominant cell type expressing S-100B IR are astrocytes of varying morphology. The morphology of the astrocytes was defined based on the size of the cell body, complexity of processes, and approximate number. *Size: *cells were defined as small, if cell bodies were less than 4 *μ*m, medium at 4–8 *μ*m, and large if the cell body was greater than 8 *μ*m. *Processes:* cells were classified as simple (no significant processes, similar to cells described as “protoplasmic,” immature or Type I), complex (many ramified processes, arranged symmetrically around the cell body, similar to “stellate” or “fibrous”), or atypical (cells with nonsymmetrical processes, such as one large think process). *Number:* cell density was defined as sparse, moderate, or dense based on their relative appearance. *Hippocampal subfields*: in general, area CA2 represented a homogenized zone between CA3 and CA1, demonstrating immunoreactivity for the types of cells seen in both regions. Thus, only areas CA1 and CA3 were analyzed. When referring to CA3 lacunosum moleculare (LAC), this represents the area of LAC which is adjacent to CA3/CA2 region. The rest of LAC is considered to be in the CA1 region. In the dentate gyrus, the morphological appearance of the supra and infrapyramidal blades of the molecular layer of the dentate gyrus was indistinguishable, and thus these are classified together. The following abbreviations are used to describe the hippocampal subfields: Pyramidal Cell Layer (PYR); Stratum radiatum (SR); Stratum oriens (SO); Lacunosum Moleculare (LAC); Dentate Molecular layers (MOL); Dentate Granule cell layer (DGN); Polymorphic zone (PMZ).
